# A pragmatic evidence-based approach to post-mortem perinatal imaging

**DOI:** 10.1186/s13244-021-01042-1

**Published:** 2021-07-15

**Authors:** Susan C. Shelmerdine, J. Ciaran Hutchinson, Celine Lewis, Ian C. Simcock, Thivya Sekar, Neil J. Sebire, Owen J. Arthurs

**Affiliations:** 1 Great Ormond Street Hospital for Children NHS Foundation Trust, London, WC1N 3JH UK; 2 UCL Great Ormond Street Institute of Child Health, London, UK; 3 Great Ormond Street Hospital NIHR Biomedical Research Centre, London, UK; 4 Population, Policy and Practice Department, UCL GOS Institute of Child Health, London, UK; 5 North Thames Genomic Laboratory Hub, Great Ormond Street Hospital, London, UK

**Keywords:** Radiology, Autopsy, Diagnostic imaging, Foetus, Pregnancy loss

## Abstract

Post-mortem imaging has a high acceptance rate amongst parents and healthcare professionals as a non-invasive method for investigating perinatal deaths. Previously viewed as a ‘niche’ subspecialty, it is becoming increasingly requested, with general radiologists now more frequently asked to oversee and advise on appropriate imaging protocols. Much of the current literature to date has focussed on diagnostic accuracy and clinical experiences of individual centres and their imaging techniques (e.g. post-mortem CT, MRI, ultrasound and micro-CT), and pragmatic, evidence-based guidance for how to approach such referrals in real-world practice is lacking. In this review, we summarise the latest research and provide an approach and flowchart to aid decision-making for perinatal post-mortem imaging. We highlight key aspects of the maternal and antenatal history that radiologists should consider when protocolling studies (e.g. antenatal imaging findings and history), and emphasise important factors that could impact the diagnostic quality of post-mortem imaging examinations (e.g. post-mortem weight and time interval). Considerations regarding when ancillary post-mortem image-guided biopsy tests are beneficial are also addressed, and we provide key references for imaging protocols for a variety of cross-sectional imaging modalities.

## Key points

- Alternatives to a standard ‘invasive’ autopsy may include less invasive alternatives such as using only post-mortem imaging (termed a ‘non-invasive autopsy’) or the addition of image-guided organ biopsies (known as a ‘minimally invasive autopsy’).
- Early gestational losses (< 20-week gestation) require specialist high-resolution imaging (e.g. micro-CT or high-field MRI) due to small foetal size.
- Post-mortem ultrasound and MRI are useful for imaging larger foetuses (> 20-week gestation), but unenhanced CT is usually unhelpful in this clinical context, due to limited intrinsic soft tissue detail.

## Background

**Fig. 1:**
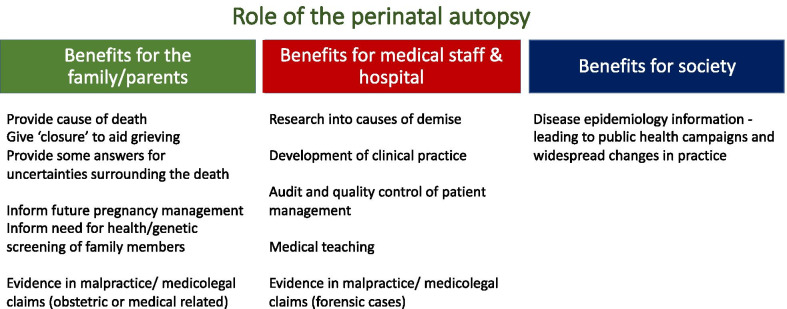
The benefits for conducting a perinatal autopsy for different stakeholders

Following the loss of a baby, autopsy is the single most useful investigation after death, yielding additional information or diagnosis in 40–70% of cases [1, 2], of which up to 50% may not have been clinically suspected [3–5]. Whilst > 90% of parents are offered an autopsy, the majority refuse this investigation, leading to low autopsy uptake rates (30–40%) [6]. Reasons for refusal include dislike of the invasive procedure, wanting to ‘protect’ the child from further harm as well as religious and cultural beliefs (Table 1) [7]. As a result, many parents do not obtain important information regarding reasons behind their pregnancy loss (and potential future pregnancy losses), and some have reported regretting their decision not to proceed with an autopsy, feeling that many questions remain unanswered [8]. There are also other benefits for performing a perinatal autopsy for the medical community and society at large, including epidemiological information, improved understanding of perinatal pathologies and quality control management (Fig. 1).

**Table 1:** Religious attitudes towards post-mortem investigation [59]

Religion	Autopsy	Tissue retention	Disposal of the body
Atheism	No prohibition	No prohibition	Burial or cremation
Christianity	No religious prohibition	No religious prohibition	Burial or cremation
Hinduism	No religious prohibition	No religious prohibition	Cremation without delay
Sikhism	No religious prohibition	No religious prohibition	Cremation without delay
Islam	Only if required by law	Only if required by law	Burial without delay
Judaism	Only if required by law	Only if required by law	Burial without delay

In many institutions, at the time of perinatal loss, parents are usually only provided with the binary option of a standard (invasive) autopsy or no further post-mortem investigation. This ‘all-or-nothing’ approach is now slowly being supplemented in some specialist centres with the choice of a ‘less invasive autopsy’ (LIA) which involves performing post-mortem imaging (instead of dissection of the body) and proceeding with minimally invasive tissue sampling (via image-guided organ biopsies) where necessary. Nevertheless, when and how to perform the most appropriate imaging for different clinical scenarios can be difficult for radiologists who infrequently encounter these situations, especially given the lack of published guidelines for reference.

This review aims to help the general and specialist paediatric radiologist understand the advantages and disadvantages of different perinatal post-mortem imaging options to allow for a more open and informed discussion with referring clinicians and bereaved parents. It is structured in a format that addresses commonly encountered queries surrounding the promise and reality of post-mortem imaging, and we provide a pragmatic, evidence-based protocol to address the majority of clinical scenarios that are likely to arise.

## Less Invasive Autopsy (LIA): What is it and how is it different to a standard autopsy?

**Fig. 2:**
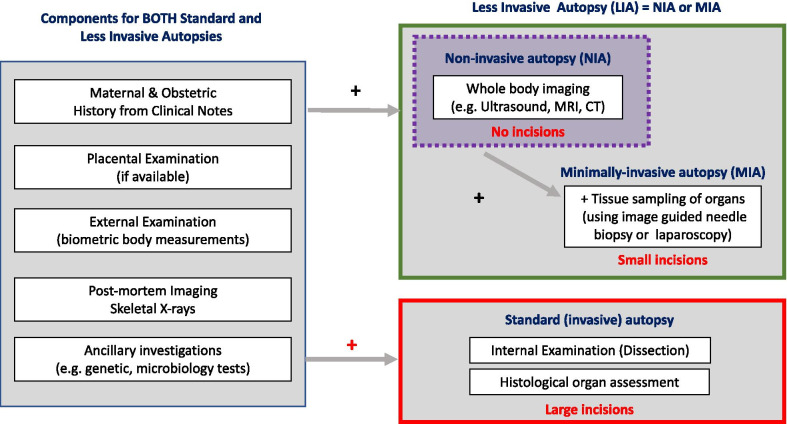
Components of different types of perinatal autopsy. A ‘less invasive autopsy’ (LIA) is an umbrella term for all procedures that use imaging instead of dissection for internal examination at autopsy. Where tissue sampling is also performed in a less invasive way (e.g. image-guided biopsy or laparoscopically assisted biopsy), the study is referred to as a ‘minimally invasive autopsy’ (MIA). Where no incisions are made to the body, and only imaging is acquired, this is termed a ‘non-invasive autopsy’ (NIA) [6]

Many parents perceive a standard ‘invasive’ autopsy to only involve the dissection of organs within their child’s body. In fact, standard autopsy consists of many additional non-invasive components, including external inspection of the body, placental examination as well as ancillary investigations such as genetic testing (Fig. 2).

‘Less invasive autopsy’ (LIA) is an umbrella term generally used to indicate any post-mortem examination where the internal examination (i.e. body dissection) is replaced with cross-sectional post-mortem imaging. The same non-invasive components (as described for the standard autopsy) are often carried out. Where tissue sampling is required (and parental consent provided), image-guided needle biopsies or a laparoscopic approach may be used via small incisions. This allows ancillary tests to be performed (e.g. genetic analysis) as well as histological assessment of targeted organs or lesions. Where image-guided tissue samples are acquired, the investigation is termed a ‘minimally invasive autopsy’ (MIA) [6].

## What is the aim of perinatal post-mortem imaging?

The reasons for perinatal losses can be broadly classified into those relating to maternal health issues (e.g. thrombophilia), placental and cord abnormalities, obstetric complications and acquired (infection) or congenital foetal anomalies [9]. Within developed countries, the commonest referral indications for post-mortem imaging relate to assessment of developmental foetal anomalies and perinatal complications (e.g. intracranial haemorrhage). Identification of these pathologies can help understand the reasons for foetal demise or better characterise antenatal imaging findings, particularly where there was a termination of pregnancy.

It is important to bear in mind when counselling parents and clinicians that despite thorough investigations, there remain a significant proportion of perinatal deaths in which the cause for foetal demise remains ‘undetermined’ despite standard autopsy [10, 11]. In addition, some pathologies, such as infection, cannot be radiologically excluded in any cases as typical findings (e.g. pulmonary consolidation) can mimic normal expected post-mortem changes [12]. This should not be used as a reason to refuse performing post-mortem imaging (more to temper any unrealistic expectations), as many parents report feeling a sense of relief and reassurance by an unremarkable result, absolving them of guilt and blame for their loss.

## What imaging modalities are best suited for perinatal post-mortem imaging?

**Fig. 3:**
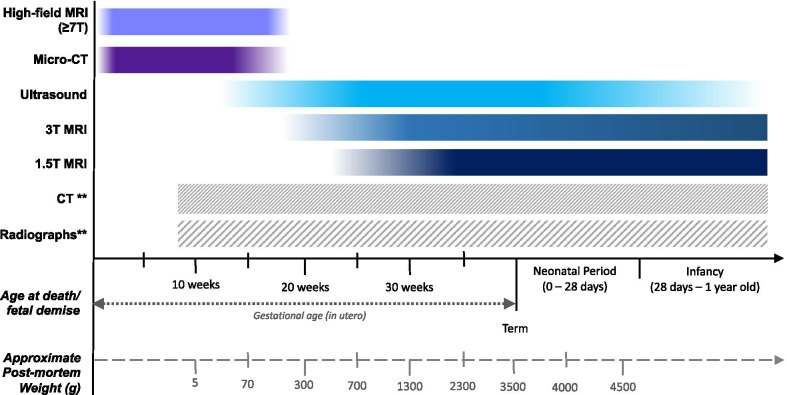
Typical estimated gestational ages and post-mortem weights where various post-mortem imaging modalities could be used to provide diagnostic quality examinations. ** Technically, radiographs and CT can be performed at any age after 8-week gestation (when the foetal skeleton beings to ossify), but in practice they are best reserved for specific clinical situations, such as for suspected skeletal abnormalities or trauma [57]

Different imaging modalities have different advantages and disadvantages according to the clinical scenario and are also gestational age dependent, largely due to issues related to image resolution. Table 2 provides a summary of factors to take into consideration when deciding which post-mortem imaging modality to conduct, and Fig. 3 provides a visual overview of which studies are most likely to be diagnostic at different gestational ages and sizes.

**Table 2:** Benefits and drawbacks of different post-mortem imaging modalities for perinatal loss

	Radiographs	Ultrasound	CT	MRI (3 T or 1.5 T)	Micro-CT	High-field MRI (7 T +)
Availability	Easily available	Easily available	Easily available	Moderate	Limited: few select centres/research facilities	Limited: few select centres/research facilities
Cost	Cheap	Cheap	Cheap	Expensive +	Same cost as CT scanner	Expensive + +
Size of foetus	Any size	Any size—although intrauterine retention time may affect image quality	Technically feasible, but poor diagnostic accuracy and lack of internal contrast from lack of body fat	Better for larger foetuses, poorer for body weight < 500 g	Up to 30 cm in length, limited by scanner bore	Similar to micro-CT
Advantages	Easy to perform, already part of routine autopsy service	Ease of access, cheap and portable Facilitates image-guided biopsies	Highest accuracy for intracranial and musculoskeletal trauma (older children; trauma)	Multiple sequences, multiplanar reconstructions	Excellent resolution and soft tissue detail Excellent bone detail without exogenous contrast	Excellent resolution and soft tissue detail No need for exogenous contrast
Drawback	No internal soft tissue detail Only useful in minority (< 5%) of cases	Operator dependent Requires a hands-on approach (radiologist)	Poor soft tissue detail due to lack of internal body fat	Availability/access may be limited Poorer resolution in smaller foetuses	Iodine contrast is required for soft tissue detail, which can cause tissue discoloration	Expensive, limited access, long scanning times (hours)
Indication:	Estimation of foetal gestational age, diagnosis of skeletal dysplasias and limb anomalies	Assessment of soft tissue/ internal organ detail	Bony injuries; trauma; consider for skeletal dysplasias or trauma (although radiographs better and cheaper)	Assessment of soft tissue/ internal organ detail	Small foetuses (< 20-week gestation) where ultrasound and 1.5 T/3 T MRI non-diagnostic	Currently research tool only

In general, for mid-second and third trimester perinatal losses (i.e. > 20-week gestation), whole-body post-mortem ultrasound (PMUS) or MRI (PMMR) is the most appropriate tools. This is in contrast to adult post-mortem imaging where CT is the commonest modality. For perinatal deaths, the lack of internal soft tissue contrast makes CT a less helpful tool [13].

Smaller foetuses, weighing less than 500 g (post-mortem bodyweight) or aged less than 18-week gestation, are more challenging to image with standard imaging technology [14], and specialist techniques are required such as high-field MRI (> 7 T) or ‘microfocus computed tomography’ (i.e. micro-CT)[15]. Local availability will largely determine what can be provided at each specialist centre, sometimes requiring a referral to be made to another centre.

Although the time interval between foetal delivery and post-mortem imaging (i.e. the ‘post-mortem interval’) has not been reported as a major factor in diagnostic post-mortem imaging quality, the degree of maceration does (relating to time between intra-uterine foetal demise and delivery, so-called intra-uterine retention time). This has been reported to be the most significant factor in acquiring a diagnostic quality post-mortem ultrasound study [16, 17] due to the degree of tissue breakdown and laxity of skull sutures. It would therefore be helpful to preferentially acquire an MRI where maceration is known to be extensive (usually when the intra-uterine retention time is estimated > 24 h).

It is therefore important to consider the following when assessing referrals for post-mortem imaging:

- What is the post-mortem weight of the foetus?
- What was the time interval between the last reported foetal movements and the delivery of the baby?
- Were there foetal abnormalities detected during the pregnancy at ultrasound or MRI?
- Has there been any previous history of perinatal loss, particularly with congenital abnormalities that could be recurrent (e.g. inheritable skeletal dysplasias)?
- Has the placenta already been examined and provided a clear cause for the perinatal loss (e.g. florid chorioamnionitis)?
- What imaging modalities are available locally; would this case require a referral to a specialist centre for post-mortem imaging?

**Fig. 4:**
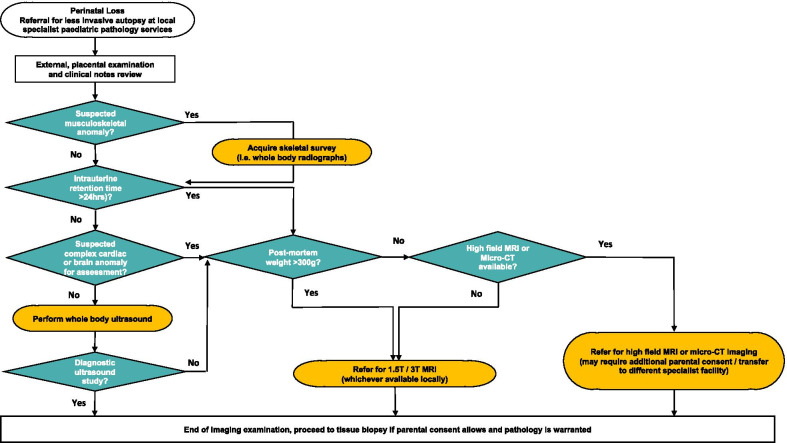
Recommended post-mortem imaging flowchart for non-invasive investigation of perinatal loss. Small foetuses present a challenge for post-mortem imaging, and care should be taken when interpreting imaging results in this cohort. A foetus weighing over 500 g provides the greatest likelihood for a diagnostic quality 1.5 T MRI study [14], and those weighing < 300 g are best suited for micro-CT or high-field MRI [32]. Where neither MRI nor micro-CT is available, ultrasound may be attempted but there is a higher likelihood of a false or non-diagnostic result [26, 58]. Foetuses weighing between 300 and 500 g have been reported to take > 7 days to iodinate and therefore delay micro-CT imaging. If available, 3 T MRI could be attempted for this foetal cohort [22]

A pragmatic, evidence-based protocol is provided in the form of a flowchart in Fig. 4, to help guide referrers and radiologists to which imaging modality would be best suited for different clinical scenarios. It is important to recognise that whilst referrers may state a gestational age for the foetus, this is not usually as helpful as knowing the post-mortem weight in determining the appropriate imaging study.

## How accurate are the different post-mortem imaging modalities?

**Fig. 5:**
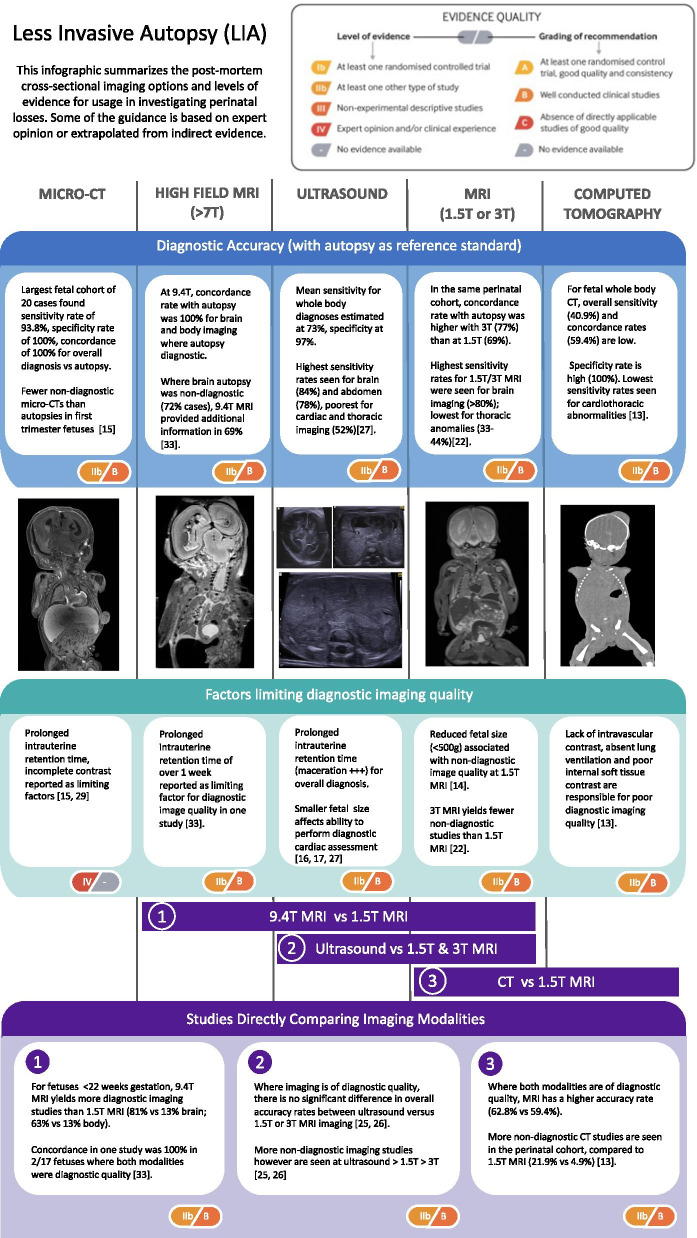
Perinatal post-mortem imaging for less invasive autopsy. This infographic summarises the perinatal post-mortem cross-sectional imaging options and levels of evidence for their usage

An infographic is provided (Fig. 5) summarising the latest published research for each imaging modality, and comparisons between different tools where available. In conducting this review of the research, we searched the PubMed, Embase and Google Scholar databases for search terms including ‘post-mortem’, ‘autopsy’ with ‘imaging’ and ‘perinatal’, ‘foetal’ or ‘neonatal’. The studies included here were chosen as being representative of the literature based on having the largest sample size population for the relevant imaging modality studied, and preference was given to systematic reviews or studies comparing two or more modalities in the same population group to enable comment for differences in diagnostic accuracy. Opinion pieces, non-human studies and case reports were excluded.

### MRI

In the largest prospective paediatric post-mortem imaging study to date (the ‘MARIAS’ study [18], including 400 children, of whom 277 (69%) were perinatal losses), there was > 90% concordance for overall diagnosis compared to standard autopsy (sensitivity of 89.7%, specificity of 95%), particularly for abnormalities of the heart, brain and musculoskeletal system. High accuracy rates have been similarly found in subsequent publications using different perinatal populations [19–21], and where available, it has been shown that performing post-mortem MRI at 3 T results in higher concordance rates with autopsy than 1.5 T MRI [22] (77% vs. 69%, respectively).

MRI has also been reported to provide clinically useful information where neuropathology was non-diagnostic [23]; however, it has now been suggested that where an (antenatal) foetal brain MRI has been performed, this is more likely to yield useful information for diagnosing complex neurological conditions [24] rather than the post-mortem MRI.

### Ultrasound

When the imaging is of diagnostic quality, ultrasound has been reported to have a similar accuracy to both 1.5 T [25] and 3 T MRI [26], with an estimated overall sensitivity of 73% and specificity 97% (based on a systematic review of 455 perinatal losses)[27]. The highest sensitivity rates were found for brain imaging (84%) and lowest for cardiothoracic abnormalities (51%). It is important to note that extensive maceration reduces the diagnostic quality of the imaging, particularly for brain imaging [16, 17]. Where there is maceration or a need to confirm and characterise an underlying cardiac anomaly, post-mortem MRI may be the more appropriate imaging modality.

### CT

In a subset of cases from the MARIAS study that underwent both 1.5 T MRI and CT (n = 82) [13], it was found that CT generated a greater number of non-diagnostic studies (22% versus 5%) and the overall accuracy rate was also lower (59% versus 63% where both CT and MRI studies were of diagnostic quality). For these reasons, CT is rarely performed in perinatal post-mortem imaging, but may be more useful in older children, particularly where there is a traumatic or forensic history [28].

### Micro-CT

Two of the largest case series published comparing foetal micro-CT with standard autopsy [15, 29], both demonstrated high sensitivity and specificity rates for overall diagnosis (94–100% sensitivity, 90–100% specificity) [15, 29]. The main drawback, however, was the requirement for tissue staining with an iodinated contrast medium which caused some residual discolouration of the foetus and tissue shrinkage [30] and can take several days for full iodination to occur, depending on the size of the foetus. There is also a current lack of availability of this tool within healthcare settings [31].

### High-field MRI

This remains a specialist tool only available in some research centres. A recent systematic review [32] only found three publications where whole body post-mortem foetal MRI was performed using a high field (7–11 T). The largest of these studies [33] (n = 17) reported complete agreement between 9.4 T MRI and standard autopsy. Contrast staining of the foetus is not required (unlike for micro-CT); however, scanning times can be lengthy taking hours in some cases.

## When would additional tissue sampling be beneficial over imaging alone?

In many cases (60% of intra-uterine deaths), the foetal death is unexplained despite even a standard ‘invasive’ autopsy [10]. Where a cause exists, this is frequently identified through non-invasive means (i.e. 38% intrauterine deaths via placental and clinical assessment) [10]. A recent publication assessing outcomes from > 5000 paediatric autopsies has shown that histological tissue sampling only provides the cause of death in a minority of perinatal cases when no clinical or macroscopic abnormality of the organ is identified, and where placental tissue was available for examination [34] (i.e. low likelihood of histological abnormality where the organ appeared normal at inspection or post-mortem imaging). Furthermore, where antenatal ultrasound and post-mortem MRI results are concordant, the additional value of an autopsy is low (< 5%) [35]. Therefore, the greatest benefit for tissue sampling is clearly where there is a structural anomaly for further investigation, and for obtaining samples for ancillary investigations.

Image guidance is preferred over ‘blind’ percutaneous needle biopsies that use surface landmarks to locate organs, as there is a low tissue targeting success rate (< 52%). Ultrasound-guided biopsies are more successful (76.1%) and can be performed via the umbilical vein mitigating incisions to the body [36]. Laparoscopically guided tissue sampling yields the highest success rates (> 80%) [37] but can be difficult to perform in small foetuses, and necessitates small incisions and more expensive equipment not commonly found in many mortuaries. It is important prior to conducting any tissue sampling that parental consent has been provided for this.

## Published protocols for paediatric and perinatal post-mortem imaging techniques

A recommended post-mortem MRI imaging protocol has been devised via an expert consensus survey conducted by the European Society of Paediatric Radiology (ESPR)[38]. An abbreviated protocol can also be followed if MRI scanner time is particularly limited [39]. A more comprehensive article on the different post-mortem MRI sequences is also provided and highly recommended [40].

The ESPR and the International Society for Forensic Radiology and Imaging (ISFRI) have published recommendations for paediatric post-mortem CT imaging [41]; however, these are typically applied to forensic childhood cases rather than perinatal deaths. Where post-mortem ultrasound is performed, two articles in Insights into Imaging describe how to conduct, report and recognise common developmental pathologies [42].

A step-by-step guide for conducting post-mortem micro-CT imaging has been published [31], and currently, high-field MRI still remains predominantly a research tool, with only a few centres describing their methodology [32, 43].

## Stakeholder perceptions of the less invasive autopsy

### How do healthcare professionals perceive the less invasive autopsy?

Healthcare professionals (e.g. obstetricians, pathologists, midwives) have reportedly found it helpful to be able to provide a greater variety of post-mortem examination options (e.g. imaging) to parents when consenting for autopsies in general [44]. Their main concerns regarding less invasive approaches relate to those of missed diagnoses and the ability for the post-mortem imaging to be provided locally.

### How do parents perceive the less invasive autopsy?

In general, acceptance rates are high. In one study, it was reported that 91% of 859 parents surveyed indicated they would consent to a less invasive autopsy over standard autopsy if they had been given the choice [45]. Almost half (46%) preferred imaging with organ tissue sampling, 31% preferred imaging alone and 14% preferred standard autopsy.

Parents valued post-mortem imaging because it allows the baby to ‘rest in peace’ and put parents ‘more at ease’, but also valued approaches where tissue samples were obtained via a small incision, as they were considered a ‘good compromise’ between the least and most invasive approaches.

Religious parental groups have also expressed support for post-mortem imaging examinations as a religiously acceptable replacement over body dissection, as long as the body can be returned swiftly for burial. Minimally invasive options were less acceptable, although preferable to the standard autopsy, and some religious parents would consider this option if there had been multiple pregnancy losses [46, 47] (Table 1).

### What is the best way to consent parents for post-mortem imaging?

It is important that consent is conducted in a sensitive and compassionate manner. Some bereaved parents will have clear views regarding their level of acceptance for invasiveness of a standard autopsy; however, a subset will be undecided. ‘Decisional drivers’ [48] include an open approach by a trusted practitioner, adequate time for deliberation and adopting an individualised approach (both in the required depth and amount of information provided). There is rarely a ‘correct’ answer, and each parent will need to feel supported in their personal patient journey [46]. It may be helpful to highlight relevant charity groups for additional emotional support.

On a practical level, permission to perform post-mortem imaging is included as part of the standard autopsy consent form at our institution to minimize additional paperwork [49]. Parental consent is also sought at the same time for the use of post-mortem images in research, teaching, audit and education. This avoids repeated, unnecessary and potentially distressing discussions with parents.

### Preparing a department for perinatal post-mortem imaging referral practice

Having a pre-defined plan of what services can be provided and how referrals can be made through multi-disciplinary team discussions are vital. Several articles on the initial experiences of other centres in developing a paediatric post-mortem imaging service have been written, which contain useful information for further reading [50–56].

Some key points to consider include:

- Identifying which imaging modalities are available locally for post-mortem imaging and which members of staff (both radiologists and radiographers/sonographers) would be comfortable to be involved in the process (e.g. vetting referrals, protocolling and reporting imaging studies).
- Clarifying the availability and procedure for external referrals to specialist centres for other post-mortem imaging modalities that are not available locally (e.g. micro-CT or high-field MRI).
- Determining whether an expedited investigation can be performed, if required (e.g. within 24 h for religious reasons)
- Availability of funding streams to maintain a post-mortem imaging service, and for the training of radiologists and radiographers in setting up such a service (e.g. attendance at courses, conferences and observership at centres which regularly carry out paediatric post-mortem imaging).
- There may be additional medicolegal requirements depending on the jurisdiction in which post-mortem imaging is being performed.

## Conclusions

Perinatal post-mortem imaging can provide a non-invasive method for death investigation, but can also aid in MIA or provide additional information if a full autopsy is needed. A variety of tools and their advantages and drawbacks are addressed in this article, with a suggested flowchart to help guide radiologists unfamiliar with the best tools to use in different perinatal death settings. Key aspects from the perinatal history include gestational age, antenatal anomalies (in particular cardiac and brain malformations) as well as more pragmatic details regarding availability of different scanners locally. Key challenges for post-mortem imaging still remain regarding local scanner availability, appropriate parental consent and funding streams.

## Data Availability

Data sharing is not applicable to this article as no datasets were generated. Summary of findings from the previously published articles is provided already in figures within this review.

## Abbreviations

CT: Computed tomography
MIA: Minimally invasive autopsy
MRI: Magnetic resonance imaging
PACS: Picture archiving and communications system
PM Micro-CT: Post-mortem microfocus computed tomography
PMCT: Post-mortem computed tomography
PMMR: Post-mortem magnetic resonance imaging
PMSS: Post-mortem skeletal survey
PMUS: Post-mortem ultrasound
